# 3D Assessment of Endodontic Lesions with a Low-Dose CBCT Protocol

**DOI:** 10.3390/dj8020051

**Published:** 2020-05-13

**Authors:** Marco Portelli, Angela Militi, Antonino Lo Giudice, Roberto Lo Giudice, Lorenzo Rustico, Rosamaria Fastuca, Riccardo Nucera

**Affiliations:** 1 Dept. of Biomedical, Dental Science and Morphological and Functional Images Dental School, Faculty of Medicine, University of Messina, 98100 Messina, Italy; amiliti@unime.it (A.M.); nino.logiudice@gmail.com (A.L.G.); rlogiudice@unime.it (R.L.G.); lorenzo.rustico@gmail.com (L.R.); riccardon@unime.it (R.N.); 2 Department of Medicine and Surgery, School of Medicine, University of Insubria, Via G. Piatti 10, 21100 Varese, Italy; rosamariaf@hotmail.it

**Keywords:** 3D imaging, low-dose cone beam, endodontic lesions

## Abstract

**Background**: Cone beam computed tomography (CBCT) is often used in different fields of dental science, especially in complex anatomical districts like the endodontic one. The aim of this study is to propose a low-dose CBCT protocol useful in cases of endodontic lesions. **Methods**: The device used was a MyRay Hyperion X9-11x5; the low dose setting of the machine was 90 Kv, 27 mAs, CTDI/Vol 2.89 mGy. The absorbed organ doses have been evaluated with an anthropomorphic phantom loaded with thermoluminescent dosimeters positioned at the level of sensitive organs like brain, bone marrow, salivary glands, thyroid, esophagus, oral mucosa, extrathoracic airways, and lymph nodes. Equivalent and effective doses have been calculated; the last one has been calculated using the recommendations approved by the Main Commission of ICRP (International Commission Radiological Protection) in March 2007. For the assessment of image quality, five senior clinicians, independent and experienced clinicians, were asked to state if CBCT scans were accurate enough to assess endodontic lesions. **Results**: The use of a low-dose CBCT acquisition produced the lowest organ dose (5.01 microSv) at the level of the esophagus. Image quality has been considered accurate enough for endodontic diagnostic needs. Conclusions: CBCT low-dose protocol can be used over the standard one in endodontic special cases because it provides a significantly lower radiation dose to the patients while ensuring good image quality. However, further studies are necessary to evaluate the opportunity of low-dose CBCT exams in endodontic clinical practice.

## 1. Introduction

3D imaging technologies have completely changed the diagnostic possibilities in dentistry, offering several advantages in different fields, among which is endodontics. Computed tomography (CT) is used when a three-dimensional evaluation is required; however, its use is limited for different reasons, and, above all, by the stochastic effects produced by ionizing radiations. Even though the spiral CT low-dose protocol was developed years ago, the main limitation of CT is the radiation dosage [1]. Cone beam CT (CBCT) machines, thanks to their limited dimensions, are ideal to be installed in a dental practice; for this reason, this apparatus has been widespread in recent years [2]. CBCT can be considered the gold standard exam in different clinical cases since it offers high-quality images, ensuring high diagnostic reliability and a good risk-benefit ratio [3]. CBCT is recommended in cases of skeletal malocclusions [4], dental anomalies [5], temporo-mandibular joint disorders [6,7], evaluation of maxillo-facial growth and development [8], anatomical assessment before temporary anchorage device (TAD) insertion [9], and management of surgical patients [10]. CBCT technology, overcoming bidimensional exam limits, could represent the gold standard in cases of particular root canal anatomy or for the assessment of endodontic lesions. The SEDENTEXCT review concluded that current evidence suggests that CBCT may have higher sensitivity for the detection of periapical lesions that conventional radiograph [11]. The use of radiographic tests has to follow the ALARA [12] principle, which states that radiological risk for the patients must be “as low as reasonably achievable”. Compared to a traditional bidimensional exam, CBCT radiation doses are higher [13], but when compared to those of a multi-slice CT, the radiation doses are considerably lower [14]. Several studies make a comparative evaluation of the radiation doses produced by CBCT, MSCT (multi-slice computed tomography), and traditional bidimensional x-ray exams [2,15,16], but protocols usable for all CBCT machines are missing [17]. Absorbed dose represents the amount of X-ray energy absorbed by a unit mass of tissue and is measured in Gray; equivalent dose instead expresses the biologic effect on a tissue produced by radiation absorption and is measured in Sievert. The effective dose indicates the radiation risk, considered as the possibility of biological consequences after radiation exposure to humans beings, and is calculated in µ-Sievert. The estimation of effective dose is obtained, translating the absorbed dose for individual organs to an equivalent dose, and then multiplied with a weighting factor defined by the International Commission on Radiological Protection (ICRP) for the specific organ. Effective dose is not a direct measurement, but a calculated value [18]. The aim of this study is to evaluate if a low-dose CBCT protocol can be accurate enough to assess endodontic lesions.

## 2. Materials and Methods

**Figure 1:**
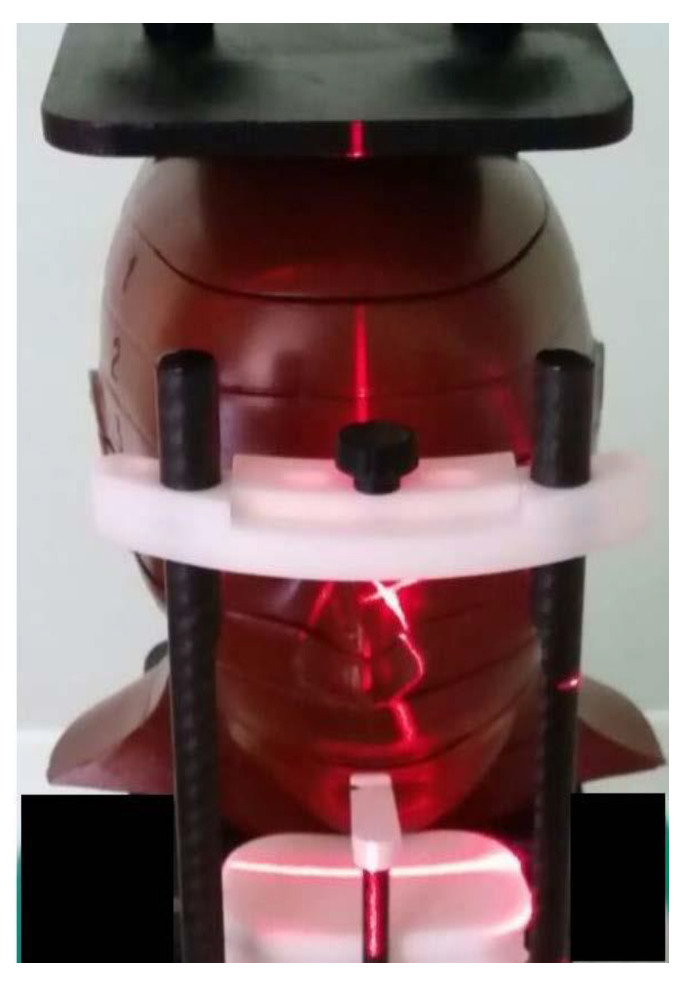
Head–neck phantom positioned in the cone beam computed tomography (CBCT).

For the study, a tissue-equivalent head-neck radiotherapy humanoid phantom Alderson Rando (The Phantom Laboratory, New York, USA) was used; an effective atomic number and a mass density of phantom exactly reproduced the muscle tissue and fat of human beings. The phantom was divided into 2.5-cm thick sections, with different 5-mm diameter holes. GR-200 A LiF thermoluminescent dosimeters (TLDs) were used and, in order to select homogeneous TLD batches, three irradiations and reading procedures were done. The TLDs were read using a RADOS TLD reader controlled by the software Dostld4.0, which can provide the glow curve in use at the Department of Healthcare Physics of the University of Messina. The readout cycle consisted of a preheat to 100 °C for 3.5 s, followed by subsequent heating at 15 s at 240 °C. The cycle was chosen after a visual examination of the glow curve from a number of different heating times and maximum temperatures. An overall time of 18.5 s was necessary for the total readout. Annealing procedure followed the standard protocol used in the Department of Healthcare Physics of the University of Messina. TLDs were calibrated by simultaneous irradiation with a conventional X-ray tube SimView 3000, with the following protocol: field 10 × 10 cm^2^, SDD (source detector distance) = 100 cm, 113 KV, 14 mm Al. Twenty four TLDs were positioned in the phantom with the following distribution: 9 TLDs at the bone marrow and surface, 5 TLDs at the salivary glands, 2 TLDs both at the brain and thyroid gland, 1 TLD at the esophagus and 5 TLDs at the other remaining tissues (oral mucosa, extrathoracic airways, lymph nodes). The machine used for the study was a Hyperion X9-11x8 (MyRay, Imola-BO, Italy) located in a private practice and offered in free use to the Dental School of the University of Messina. The standard setting of the apparatus was 90 Kv, 36 mAs, CTDI/Vol 4.09 mGy; the original low-dose protocol proposed in the present study instead was set at 90 Kv, 27 mAs, CTDI/Vol 2.89 mGy. The phantom was positioned according to the manufacturer’s specifications, following the reference lines and headrests (Figure 1).

The field of view (FoV) of the apparatus was 11 inches; this allowed us to obtain images from the whole maxilla and mandible bones, including the Temporomandibular joint TMJ area. The calculation of the effective dose has been obtained by multiplying the organ doses to a risk weighting factor that is influenced by organ sensitivity [19]. This measurement has been calculated as follows: E = WT × HT, where E is the product between the ICRP’s tissue-weighting factor (WT) and the type or tissue or body and the human-equivalent dose for tissue (HT). The tissue-weighting factor expresses the contribution of each tissue or organ to the overall risk. This dose was expressed in MicroSv. In the present study, we used the weighting factors proposed by the ICRP in 2005 and approved by the Main Commission of ICRP in March 2007 [20]. Image quality evaluation was performed on the basis of the possibility to easily acquire the information useful to assess root canal anatomy and/or endodontic lesions and considering the level of tissue contrast, image sharpness, and overall subjective impression. Five senior clinicians were asked to judge if image rendering (tissue contrast, image sharpness, and overall subjective impression) was accurate enough to assess endodontic lesions and root canal anatomy [21].

## 3. Results

Descriptive statistics, including mean value and standard deviation, were calculated for the absorbed (Table 1), equivalent (Table 2), and effective doses (Table 3). The lowest effective dose was registered at the level of the esophagus (0.19 µ SV), while the highest one was received by oral mucosa (41.545 µ SV).

**Figure 2:**
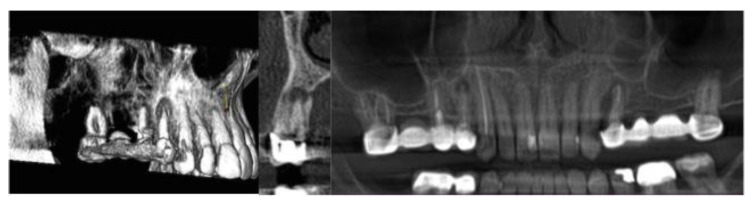
Maxillary apical granuloma in 3D, Sagittal, and Panorex views.

**Figure 3:**
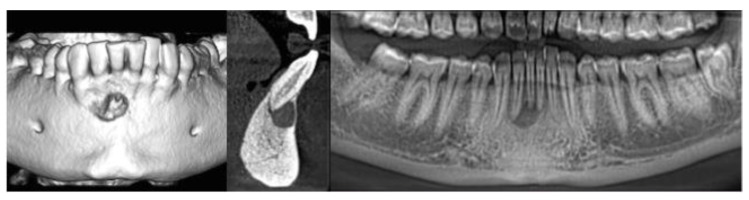
Mandibular periapical cyst in 3D, Sagittal, and Panorex views.

The assessment of image quality, performed independently by five experienced endodontists, was done, attributing individual scores ranging from 0 (very poor) to 10 (very good) [22] to the same images of periapical endodontic lesions (Figure 2 and Figure 3) acquired with the same CBCT apparatus used for dosimetric evaluation. Image quality assessment was performed on ten CBCT exams acquired between January and November 2019 and related to endodontic osteolytic lesions; age, gender, and clinical characteristics of patients are reported in Table 4. For the intra-examiner calibration, every experienced endodontist repeated the image quality assessment four times every 48 h; concordance between the sets of assessment was quite high (0.986 for the intra-examiner calibration, 0.980 for the inter-examiner calibration) and, in all cases, statistically significant (*p* < 0.001). Image quality assessment scores are reported in Table 5.

## 4. Discussion

The endodontic district is a complex anatomical region with quite a variable anatomy. In several clinical conditions, bidimensional images can provide sufficient information for diagnostic needs; however, sometimes a 3D imaging is necessary. The use of radiographic tests in dentistry should always follow the ALARA principle, which states that the radiation dose to patients must be “as low as reasonably achievable” [12]. The image quality obtained with the low dose CBCT protocol proposed in this study turned out to be widely sufficient for endodontic diagnostic needs. Ludlow et al. [16], in a comparative evaluation of different CBCTs machines with different fields of view (FoV), demonstrated that the radiological risk for patients increases with a higher FoV. The use of a CBCT technology, even if a low-dose protocol is adopted, cannot be used routinely in endodontic practice. Therefore, in maxillo-facial radiology, an accurate evaluation between the radiological risk and the diagnostic information is necessary to the clinician. When additional information is needed, such as in cases of dental anomalies [5], temporo-mandibular joint disorders [6,7], evaluation of maxillo-facial growth and development [8], anatomical assessment before TADS insertion [9], and the management of surgical patients [10], the use of CBCT can be recommended. Advanced CBCT software applications can also be used to perform particular anatomical evaluations and to perform superimpositions [23]. The side effects of ionizing radiations are the main limitations for a routine use of CBCT in clinical practice. Even if the effective dose of CBCT is significantly lower than other CT examinations involving different anatomical districts, there is concern about radiation dose for CBCT exams in dental practice. This concern is related to the high frequency of dental examinations and to the possibility of close exam repetition in the same patient, especially in cases of evolutionary lesions, such as the endodontic ones [20], or if it is necessary to evaluate treatment evolution [24]. For this reason, it is important to optimize acquisition parameters and to minimize the related radiological risk. Many studies compared the effective doses produced by multi-slice computed tomography (MSCT) and the CBCT apparatus. In a study performed by Lubele et al. in 2009, CBCT doses are about eight to ten times lower than MSCT machine ones; however, it must be considered that CBCT protocol is very heterogeneous, and this fact is always well-considered [15]. The CBCT Guidelines for dental radiology proposed by different scientific societies like the American Association of Endodontics 2015 [25] and the European Society of Endodontology 2014 [26] suggest that CBCT should be performed only when the diagnostic needs cannot be sufficiently achieved with conventional radiography. The aim of the present study is to demonstrate that a low-dose CBCT protocol can provide an image quality sufficient to the diagnostic needs, with a significant reduction of the effective dose to the organ, in accordance with the ALARA principles. The assessment of image quality performed independently by five experienced endodontists provided a mean score of 7.0. According to the results obtained in the present study, it is possible to state that the proposed CBCT low-dose protocol offers the possibility of obtaining good quality of images, with a significant reduction of radiation dose to the patient, in cases of root canal anatomy investigation [27], dental anomalies [28], periapical lesions assessment [29], and in any endodontic clinical condition that requires an accurate radiological investigation [30,31].

## 5. Conclusions

CBCT low-dose protocols should be preferred over the standard ones because they provide an image quality sufficient for the diagnostic needs with a significantly lower radiation dose to the patients. Low-dose CBCT exams are strictly indicated in clinical cases where a three-dimensional evaluation is necessary. CBCT low-dose investigations are justified in cases of periapical endodontic lesions when the complex and variable anatomy of the periapical district may occur [30]. However, further studies are necessary in order to obtain a high level of evidence of the possibility to perform CBCT investigations in endodontic practice as an alternative to the traditional bidimensional investigations [31,32,33,34].

## Figures and Tables

**Table 1:** Descriptive statistics for absorbed dose values.

ABSORBED DOSE (mGy)				
	MIN	MAX	MEDIUM	DS
Bone Marrow	0.10	0.16	0.13	0.03
Salivary Glands	0.40	0.47	0.0435	0.035
Brain	0.01	0.11	0.06	0.05
Tyroid	0.10	0.12	0.11	0.01
Esophagus	0.04	0.05	0.45	0.005
Oral Mucosa	0.32	0.36	0.34	0.02
Extra-Toracic Airway	0.14	0.16	0.15	0.009
Lymphnodes	0.21	0.22	0.215	0.005
Skin	0.1052	0.1061	0.1056	0.00045
Muscle	0.21	0.22	0.215	0.005
Bone Surface	0.10	0.16	0.13	0.03

**Table 2:** Descriptive statistics for equivalent dose values.

EQUIVALENT DOSE (µ SV)				
	MIN	MAX	MEDIUM	DS
Bone Marrow	17.52	26.63	21.89	4.555
Salivary Glands	403.97	477.68	440.825	36.885
Brain	112.83	119.58	116.205	3.375
Tyroid	100.46	123.01	111.73	11.275
Esophagus	4.43	5.59	5.01	0.58
Oral Mucosa	326.46	366.08	346.27	19.81
Extra-Toracic Airway	149.88	162.19	156.035	6.154
Lymphnodes	10.56	11.20	10.88	0.32
Skin	5.26	5.31	5.285	0.024
Muscle	10.56	11.20	10.88	0.32
Bone Surface	34.99	53.18	44.085	9.095

**Table 3:** Descriptive statistics for effective dose values.

EFFECTIVE DOSE (µ SV)				
	MIN	MAX	MEDIUM	DS
Bone Marrow	0.17	0.26	0.215	0.045
Salivary Glands	4.039	4.77	4.4045	0.3655
Brain	0.38	0.45	0.415	0.035
Tyroid	4.01	4.92	4.465	0.455
Esophagus	0.17	0.22	0.195	0.025
Oral Mucosa	39.17	43.92	41.545	2.375
Extra-Toracic Airway	17.98	19.46	18.72	0.74
Lymphnodes	1.26	1.34	1.3	0.040
Skin	0.052	0.053	0.0525	0.0005
Muscle	1.26	1.34	1.3	0.040
Bone Surface	0.34	0.53	0.435	0.095

**Table 4:** Age, gender, and clinical characteristics of the patients.

Patient	Age	Gender	Clinical Characteristics
1	46	Female	Apical Granuloma
2	31	Female	Periapical Cyst
3	56	Male	Apical Granuloma
4	49	Male	Apical Granuloma
5	27	Male	Periapical Cyst
6	52	Female	Periapical Cyst
7	48	Male	Apical Granuloma
8	61	Male	Periapical Cyst
9	43	Female	Apical Granuloma
10	39	Male	Apical Granuloma

**Table 5:** Image quality evaluation scores.

IMAGE QUALITY EVALUATION SCORES	
Endodontist I	7
Endodontist II	6
Endodontist III	7
Endodontist IV	8
Endodontist V	7
MEAN VALUE	7.0

## References

[1] Cordasco G., Portelli M., Militi A., Nucera R., Lo Giudice A., Gatto E., Lucchese A. (2013). Low-dose protocol of the spiral CT in orthodontics: Comparative evaluation of entrance skin dose with traditional X-ray techniques. Prog. Orthod..

[2] Ludlow J.B., Ivanovic M. (2008). Comparative dosimetry of dental CBCT devices and 64-slice CT for oral and maxillofacial radiology. Oral. Surg. Oral. Med. Oral. Pathol. Oral. Radiol. Endod..

[3] Yammamoto K., Ueno K., Seo K., Shinohara D. (2003). Development of dento-maxillofacial cone beam x-ray computed tomography system. Orthod. Craniofac. Res..

[4] Fastuca R., Lorusso P., Lagravère M.O., Michelotti A., Portelli M., Zecca P., D’Antò V., Militi A., Nucera R., Caprioglio A. (2017). Digital evaluation of nasal changes induced by rapid maxillary expansion with different anchorage and appliance design. BMC Oral. Health.

[5] Militi D., Militi A., Cutrupi M.C., Portelli M., Rigoli L., Matarese G., Salpietro D.C. (2011). Genetic basis of non syndromic hypodontia: A DNA investigation performed on three couples of monozygotic twins about PAX9 mutation. Eur. J. Paediatr Dent..

[6] Bartolucci M.L., Marini I., Bortolotti F., Impellizzeri D., Di Paola R., Bruschetta G., Crupi R., Portelli M., Militi A., Oteri G. (2018). Micronized palmitoylethanolamide reduces joint pain and glial cell activation. Inflamm. Res..

[7] Portelli M., Gatto E., Matarese G., Militi A., Catalfamo L., Gherlone E., Lucchese A. (2015). Unilateral condylar hyperplasia: diagnosis, clinical aspects and operative treatment. A case report. Eur. J. Paediatr Dent..

[8] Portelli M., Matarese G., Militi A., Nucera R., Triolo G., Cordasco G. (2009). Myotonic dystrophy and craniofacial morphology:clinical and instrumental study. Eur. J. Paediatr Dent..

[9] Portelli M., Militi A., Nucera R., Cicciù M., Gherlone E., Lucchese A. (2016). Orthodontic management of missing lateral incisor by miniscrew-anchored device. Minerva Stomatol..

[10] Staderini E., Patini R., Guglielmi F., Camodeca A., Gallenzi P. (2019). How to Manage Impacted Third Molars: Germectomy or Delayed Removal? A Systematic Literature Review. Medicina.

[11] SEDENTEXCT Project. www.sedentex.eu/files/radiation_protection.

[12] Berkhout W.E. (2015). The ALARA-principle. Backgrounds and enforcement in dental practices. Ned. Tijdschr. Tandheelkd..

[13] Swennen G.R.J., Schutyser F. (2006). Three-dimensional cephalometry: Spiral multi-slice vs cone-beam computed tomography. Am. J. Orthod. Dentofac. Orthop..

[14] Verhelst P.J., Verstraete L., Shaheen E., Shujaat S., Darche V., Jacobs R., Swennen G., Politis C. (2020). Three-dimensional cone beam computed tomography analysis protocols for condylar remodelling following orthognathic surgery: A systematic review. Int. J. Oral. Maxillofac. Surg..

[15] Loubele M., Bogaerts R., Van Dijck E., Pauwels R., Vanheusden S., Suetens P., Marchal G., Sanderink G., Jacobs R. (2009). Comparison between effective radiation dose of CBCT and MSCT scanners for dentomaxillofacial applications. Eur. J. Rad..

[16] Ludlow J.B., Timothy R., Walker C., Hunter R., Benavides E., Samuelson D.B., Scheske M.J. (2015). Effective dose of dental CBCT a meta analysis of published data and additional data for nine CBCT units. Dentomaxillofac Radiol..

[17] Mc Guigan M.B., Dunca H.F., Horner K. (2018). An analysis of effective dose optimization on image quality and diagnostic efficacy relating to dental cone beam computed tomography (CBCT). Swed. Dent. J. Suppl..

[18] Fisher D.R., Fahey F.H. (2017). Appropriate Use of Effective Dose in Radiation Protection and Risk Assessment. Health Phys..

[19] Recommendations of the International Commission on Radiological Protection. Draft document. https://www.icrp.org.

[20] Portelli M., Militi A., Lo Giudice A., Lo Giudice R., Fastuca R., Ielo I., Mongelli V., Lo Giudice G., Lucchese A., Nucera R. (2018). Standard and Low-Dose cone beam computer tomography protocol for orthognatodonic diagnosis: A comparative evaluation. J. Biol. Regul. Homeost. Agents.

[21] Caccianiga G., Lo Giudice A., Paiusco A., Portelli M., Militi A., Baldoni M., Nucera R. (2019). Maxillary orthodontic expansion assisted by unilateral alveolar corticotomy and low-level-laser therapy: A novel approach for correction of posterior unilateral cross-bite in adults. J. Lasers Med Sci..

[22] Staderini E., De Luca M., Candida E., Rizzo M.I., Rajabtork Zadeh O., Bucci D., Zama M., Lajolo C., Cordaro M., Gallenzi P. (2019). Lay People Esthetic Evaluation of Primary Surgical Repair on Three-Dimensional Images of Cleft Lip and Palate Patients. Medicina.

[23] Staderini E., Guglielmi F., Cornelis M.A., Cattaneo P.M. (2019). Three-dimensional prediction of roots position through cone-beam computed tomography scans-digital model superimposition: A novel method. Orthod. Craniofacial Res..

[24] Schloss T., Sonntag D., Kohli M.R., Setzer F.C. (2017). A Comparison of 2-and 3-dimensional Healing Assessment after Endodontic Surgery Using Cone-beam Computed Tomographic Volumes or Periapical Radiographs. J. Endod..

[25] (2015). AAE and AAOMR Joint Position Statement: Use of Cone Beam Computed Tomography in Endodontics 2015 Update. Oral. Surg. Oral. Med. Oral. Pathol. Oral. Radiol..

[26] Patel S., Durack C., Abella F., Roig M., Shemes H., Lambrechts P., Lemberg K. (2014). European Society of Endodontology position statement: The use of CBCT in Endodontics. Inter. Endod. J..

[27] Patel S., Patel R., Foschi F., Mannocci F.J. (2019). The Impact of Different Diagnostic Imaging Modalities on the Evaluation of Root Canal Anatomy and Endodontic Residents’ Stress Levels: A Clinical Study. J. Endod..

[28] Vitale C., Militi A., Portelli M., Cordasco G., Matarese G. (2009). Maxillary Canine-First Premolar transposition in the permanent dentition. J. Clin. Orthod..

[29] Weber M.T., Stratz N., Fleiner J., Schulze D., Hannig C. (2015). Possibilities and limits of imaging endodontic structures with CBCT. Swiss. Dent. J..

[30] Mohammadi Z., Giardino L., Palazzi F., Shalavi S., Alikhani M.Y., Giudice G.L., Davoodpour N. (2013). Effect of sodium hypochlorite on the substantivity of chlorhexidine. Int. J. Clin. Dent..

[31] Lo Giudice G., Lizio A., Lo Giudice R., Centofanti A., Rizzo G., Runci M., Alibrandi A., Cicciù M. (2016). The effect of different cleaning protocols on post space: A SEM study. Int. J. Dent..

[32] Kruse C., Spin-Neto R., Reibel J., Wenzel A., Kirkevang L.L. (2017). Diagnostic validity of periapical radiography and CBCT for assessing periapical lesions that persist after endodonticsurgery. Dentomaxillofac Radiol..

[33] Lofthag-Hansen S., Huumonen S., Gröndahl K., Gröndahl H.G. (2007). Limited cone-beam CT and intraoral radiography for the diagnosis of periapical pathology. Oral. Surg. Oral. Med. Oral. Pathol. Oral. Radiol. Endod..

[34] Lo Giudice A., Fastuca R., Portelli M., Militi A., Bellocchio M., Spinuzza P., Briguglio F., Caprioglio A., Nucera R. (2017). Effects of rapid vs. slow maxillary expansion on nasal cavity dimensions in growing subjects: A methodological and reproducibility study. Eur. J. Paediatr Dent..

